# A novel dominant *GJB2* (DFNA3) mutation in a Chinese family

**DOI:** 10.1038/srep34425

**Published:** 2017-01-19

**Authors:** Hongyang Wang, Kaiwen Wu, Lan Yu, Linyi Xie, Wenping Xiong, Dayong Wang, Jing Guan, Qiuju Wang

**Affiliations:** 1 Chinese PLA Institute of Otolaryngology, Chinese PLA General Hospital, Medical School of Chinese PLA, Beijing, 100853, China

## Abstract

To decipher the phenotype and genotype of a Chinese family with autosomal dominant non-syndromic hearing loss (ADNSHL) and a novel dominant missense mutation in the *GJB2* gene (DFNA3), mutation screening of *GJB2* was performed on the propositus from a five-generation ADNSHL family through polymerase chain reaction amplification and Sanger sequencing. The candidate variation and the co-segregation of the phenotype were verified in all ascertained family members. Targeted genes capture and next-generation sequencing (NGS) were performed to explore additional genetic variations. We identified the novel *GJB2* mutation c.524C > A (p.P175H), which segregated with high frequency and was involved in progressive sensorineural hearing loss. One subject with an additional c.235delC mutation showed a more severe phenotype than did the other members with single *GJB2* dominant variations. Four patients diagnosed with noise-induced hearing loss did not carry this mutation. No other pathogenic variations or modifier genes were identified by NGS. In conclusion, a novel missense mutation in *GJB2* (DFNA3), affecting the second extracellular domain of the protein, was identified in a family with ADNSHL.

Mutations in the gene *GJB2*, which encodes the gap junction protein connexin 26, play a key role in hereditary hearing loss and represent the most common cause of non-syndromic hearing loss^1^. Variations in *GJB2* may cause either autosomal dominant or recessive non-syndromic hearing loss (NSHL) as well as syndromic hearing loss (SHL). The inheritance pattern is ultimately determined by how the variation affects the expression and function of the connexin 26 protein. More than two hundred mutations in *GJB2* (DFNB1, MIM220290) have been found to be related to autosomal recessive non-syndromic hearing loss^2^, whereas only nineteen *GJB2* mutations have been associated with hereditary autosomal dominant non-syndromic deafness (DFNA3, MIM601544). Previous studies of dominant *GJB2* mutations are rare and have been limited to cases or simplex families. In this study, via traditional PCR and Sanger sequencing, a novel *GJB2* heterozygous mutation was identified in a Chinese family with autosomal dominant non-syndromic hearing loss (ADNSHL).

## Results

### Clinical description

**Figure 1:**
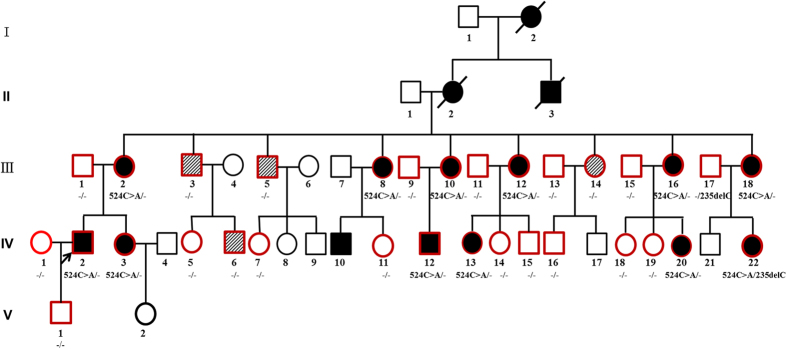
Pedigree of Family 304. Filled symbols for males (squares) and females (circles) represent affected individuals, and empty symbols represent unaffected individuals. An arrow denotes the propositus. Symbols with sloping grain indicate individuals with noise exposure history who are not mutation carriers but do present with hearing loss. Symbols with red frame indicate members who are recruited in this study, and *GJB2* gene testing results are listed below the symbols, “524C > A/−” donotes the individual with one c.524C > A variation; “−/−” represents the individual with no GJB2 variation; “524C > A/235delC” represents the individual with compound heterozegous mutation of c.524C > A and c.235delC.

**Figure 2:**
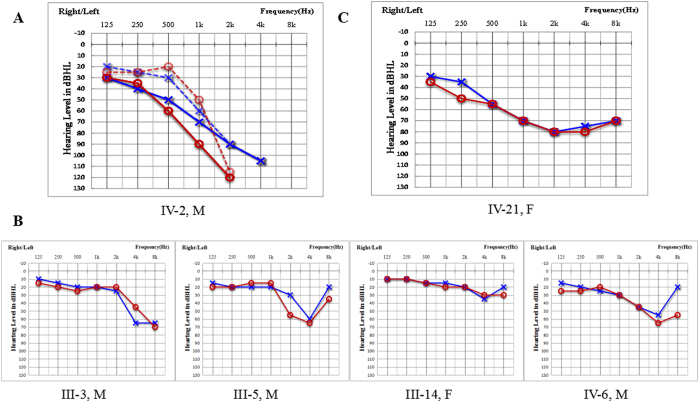
Audiograms of both ears from affected subjects in Family 304. M, male; F, female. (**A**) The dashed line represents the audiograms detected in 2004 when the propositus (IV:2) was 26 years old. Audiological examination with solid lines was performed in 2013. (**B**) Audiograms of family members with noise-induced hearing impairment. (**C**) Audiogram of subject (IV:22) who showed much more severe hearing loss. Symbols “o” and “x” denote air conduction pure-tone thresholds at different frequencies in the right and left ear. dB, decibels; Hz, Hertz.

From Family 304, a total of 32 family members, including 16 clinically affected and 16 unaffected individuals, were included in this study (Fig. 1). Among the 16 affected cases, 4 subjects had explicit noise exposure history. In this family, the 12 affected members without noise exposure history showed symmetrical and bilateral non-syndromic sensorineural hearing loss, but no clear onset age was described (although the ages of identification were generally within childhood). The propositus (IV:2) was a 26-year-old male with bilateral progressive hearing loss when he first visited our outpatient clinic in 2004. The hearing impairment initially presented at high frequencies. He showed moderate or mild hearing loss at 0.5 kHz and 1 kHz and severe or profound hearing loss at 2 kHz, 4 kHz and 8 kHz. Nine years later, the threshold had increased to approximately a 10–20 dB hearing level (HL) at 0.5 kHz–1 kHz in the left ear and 10–40 dB HL at 0.5 kHz–4 kHz in the right ear (Fig. 2A). Auditory brainstem responses (ABR) could not be evoked in both ears, and distortion product otoacoustic emissions (DPOAEs) were absent at all frequencies (Supplementary Fig. S1). Speech recognition of the propositus was 40% and 16% in the left and right ears, respectively, with an acoustic stimulus intensity of 100 dB HL in 2016. Some patients in this family reported accompanied tinnitus but no vestibular symptoms or signs (Table 1). The caloric tests and the amplitude of the cervical vestibular evoked myogenic potential (cVEMP) were normal. High-resolution computed tomography (HRCT) of the temporal bone in the propositus showed normal inner ear structures. Except for the subjects with noise exposure history (Fig. 2B), the other affected members had no other history of exposure that might account for their hearing impairment. No skin phenotypes (Supplementary Fig. S2) or other related systemic findings were identified through examination of medical histories or physical examination.

One of the affected members, IV:22 (14 years old), showed congenital severe hearing loss affecting all frequencies instead of only the high frequencies at an early age (Fig. 2C). The pure tone audiometry (PTA) of IV:22 was 70 dB HL in the left ear and 71.25 dB HL in the right ear in 2013.

### Mutation detection and analysis

A C > A substitution in exon 2 of the *GJB2* gene was identified in the propositus, which resulted in a proline to histidine change (p.P175H) in the translated sequence. This proline is located in the second extracellular loop region (EC2) of connexin 26 and is conserved among various species. Sanger sequencing confirmed the co-segregation of c.524C > A with the disease phenotype in Family 304 (Supplementary Fig. S3). The mutation was not detected in either the unaffected members of the family or those with noise-induced hearing loss, whereas all of the affected members except for the noise-induced cases carried the mutation. The genotype frequency, as reported in dbSNP137, HapMap, the 1000 Genomes Project and the local dataset, was less than 0.001 (0.000). The mutation occurred at highly conserved amino acids (Supplementary Fig. S4) and was predicted to be deleterious with the PolyPhen 2, Mutationtaster and SIFT programs. On the basis of these results, the phenotypes of the family, and the ACMG standards and guidelines, the variation identified in this study is pathogenic according to the standards of PVS1, PS4, PM1, PM2, PM5, PP1, PP3 and PP4^3^.

The *GJB2* c.524C > A variation was not identified in the 703 genomic DNA samples from a panel of affected individuals or in the 100 control genomic DNA samples from a panel of unaffected individuals. Targeted genes capture and next-generation sequencing (NGS) did not reveal any other possible disease-causing variations.

### Literature review

Our search of the literature identified 434 records in total. After duplicates were removed, 282 titles and abstracts were screened, and 46 publications were selected for full-text review. After full-text review, 43 articles remained for further consideration for data extraction. A total of 19 *GJB2*-associated DFNA3 mutations were summarized (Table 2) and included some mutations that account for both NSHL and SHL, such as p.R75W, p.R75Q, and p.R184Q.

## Discussion

In this study, we identified a novel dominantly inherited c.524C > A mutation, which leads to p.P175H conversion in the *GJB2* gene, in a Chinese family with ADNSHL, which is predictive of a decrease in the age of diagnosis and an increase in severity of hearing impairment in successive generations. In addition, we targeted 307 genes by gene capture and high-throughput sequencing and identified no other genetic variations. Unlike *GJB2* recessive mutations in which the spectrum and phenotype-genotype correlations have been analyzed clearly, few studies of *GJB2* dominant mutations have been reported. In addition, approximately two-thirds of dominant *GJB2* mutations cause syndromic hearing loss associated with diverse skin disorders, whereas only the remaining one-third of mutations cause ADNSHL^4^. To date, more than thirty dominant mutations of *GJB2* have been identified worldwide (The Human Gene Mutation Database, http://www.hgmd.cf.ac.uk), among which only 19 non-syndromic pathogenic variations have been described. However, the majority of these pathogenic variations have been described only in single families or in simplex cases^2^,^5^. Although the molecular mechanism underlying the effects of the mutation is unclear, identifying more of these variations may provide additional opportunities to decipher the pathogenic mechanism underlying hearing loss. In addition, these findings may have important implications for genetic counseling and clinical management in affected families.

We performed genotype-phenotype correlation analysis of the dominant *GJB2* mutation in this family. Most of the dominant *GJB2* mutations caused post-lingual, progressive sensorineural hearing loss that initially affected only the ability to hear high frequencies^6^. The affected members had no noise exposure history. There were also some dominant mutations related to pre-lingual hearing impairment (Table 2). Dominant *GJB2* mutations related to syndromic hearing loss and skin manifestations were excluded in Family 304. Compared with the results of previous studies in which subjects with bilateral *GJB2* mutations showed normal caloric responses and significantly lower cVEMP amplitudes, the vestibular function assessed by caloric tests and cVEMP were normal in this study^7^. Notably, hearing loss is an etiologically heterogeneous trait that is related to many genetic and environmental causes^8^. In this ADNSHL family with 16 affected members at the time of study, 11 affected members had consistent phenotypes (high-frequency hearing impairment) and genotypes (*GJB2* c.524C > A mutation carriers). In addition, 4 subjects with noise-induced hearing impairment had an explicit noise exposure history and classic noise-induced hearing loss audiograms indicating that the hearing threshold at 4 kHz was affected, and had no *GJB2* mutations; one patient (IV:22) showed much more severe hearing loss and compound heterozygosity for *GJB2* (c.524C > A/235delC). For IV:22, who showed much more severe hearing impairment than did other affected members at an early age, the compound mutations 524C > A and c.235delC were identified. The *GJB2* 235delC mutation is the most frequently known recessive mutation in some East Asian groups. The severity of the hearing phenotype associated with dominant *GJB2* mutations may be modified by additional recessive mutations in *GJB2,* and this mechanism has also been supported by studies in other families with dominant *GJB2* mutations^6^,^9^,^10^. Additional functional studies may provide further information on this phenomenon.

A complete gap junction channel consists of two hemichannels (connexons), each of which is comosed of six connexin subunits^11^ sharing a common topology consisting of four transmembrane domains (TM1–TM4), two extracellular loops (EC1 and EC2), a single intracellular loop (IC), and cytoplasmic amino- and carboxy-terminal domains. Connexin 26, which is expressed in the fibrocytes of the spiral ligament and spiral limbus, the basal cells of the stria vascularis, and the supporting cells in the Corti, plays a crucial role in K^+^ homeostasis and intracellular signaling in the inner ear^12^. Most of the dominant mutations occur in the highly conserved first extracellular loop (E1) of connexin 26, which is critical for voltage gates and connexon-connexon docking^4^. The mutation c.524C > A identified in this study is located in the second extracellular loop (E2) region of connexin 26, which plays a crucial role in interacting with other connexin molecules in the same connexon. This change in the EC2 domain may disturb the local conformation, thereby interfering with docking to the partner connexin and influencing the connexon-connexon interaction. There are six other described *GJB2* dominant mutations in the EC2 domain: p.M163V, p.M163L, p.A171T, p.P175H, p.D179N and p.R184Q (Table 2). A previous functional study of DFNA3 has shown that haplotype insufficiency is not sufficient to cause hearing impairment, whereas the dominant negative effect may be the mechanism underling *GJB2* dominant mutation-related hearing loss^13^,^14^. The mutation identified in Family 304 may also act in a dominant-negative fashion.

Genetic diagnosis and counseling have played increasingly important roles in clinical practice. Establishing a genetic diagnosis can alleviate parental guilt and anxiety, lay a foundation for future genetic counseling and provide prognostic information^15^. The offspring of an affected individual with the *GJB2* mutation in Family 304 had a 50% chance of inheriting the altered gene, except for IV:22 and the four noise-induced hearing loss members. It is also necessary for the spouse of IV:22 to receive genetic testing for *GJB2* before providing genetic counselling. For now, it has been suggested that this teenage girl, whose speech recognition was poor, wear two hearing aids. Newborn concurrent hearing and genetic screening^16^ are recommended for all of the offspring of gene mutation carriers to provide early diagnosis and intervention. Prenatal testing for pregnancies at increased risk is also possible; the mutation carriers can also choose preconception testing and diagnosis of their own accord^15^.

In summary, we identified a novel DFNA3 mutation in a Chinese family with ADNSHL and performed phenotype-genotype correlation analysis. The identification of additional dominant mutations in *GJB2* further confirmed its key role in genetic hearing loss. Moreover, genetic diagnosis and counseling should be provided to patients if necessary.

## Methods and Materials

### Ethics statement

The study was approved by the Committee of Medical Ethics of the Chinese People’s Liberation Army (PLA) General Hospital. Written informed consent from all the participants in the family were obtained. The methods were performed in accordance with the approved guidelines.

### Family recruitment and clinical evaluations

A five-generation family (Family 304) with 32 members with segregating ADNSHL was identified by the Institute of Otolaryngology, Chinese PLA General Hospital (Fig. 1). Personal or family medical reports of hearing loss, tinnitus, vestibular symptoms and other clinical abnormalities, particularly epidermal abnormalities of the participants, were identified by a team of experienced physicians and audiologists. Audiometric evaluations included audiogram, ABR, DPOAE and speech recognition. PTA was calculated as the average of the hearing threshold at 0.5, 1, 2 and 4 kHz for the better-hearing ear of each affected subject. Children under six years old were evaluated by ABR, 40 Hz AERP (auditory event-related potential). The severity of hearing impairment was defined as mild (26–40 dB HL), moderate (41–55 dB HL), moderately severe (56–70 dB HL), severe (71–90 dB HL) or profound (>90 dB HL). HRCT was also performed on the propositus to verify whether the family members had other complications other than hearing disorders. The propositus was examined through caloric testing and cVEMP testing to obtain data on semicircular canal function and otolithic function, respectively.

### Sanger sequencing

Genomic DNA was extracted from whole blood samples using a Blood DNA kit according to the standard protocol (TIANGEN BIOTECH, Beijing, China). PCR and Sanger sequencing were performed on the propositus and then on all available members from Family 304 to determine whether the potential mutation the in causative gene co-segregated with the disease phenotype in the family. The direct PCR products were sequenced using BigDye terminator v3.1 cycle sequencing kits (Applied Biosystems. Foster City, CA, USA) and analyzed using an ABI 3700XL Genetic Analyzer. The primer sequences and PCR cycles used are provided in Supplementary Fig. S5.

### Mutation analysis

To examine whether other genetic defects were involved, targeted multi-gene capture and high-throughput sequencing were performed on the propositus and his mother (III:2), including known and candidate hearing loss-associated genes^17^.

A total of 703 ethnically matched subjects with sensorineural hearing loss were also examined by Sanger sequencing for the whole sequence of *GJB2* to determine whether the variation identified in this study was recurrent. One hundred ethnically matched normal individuals comprised the control genomic DNA sample group. BLAST was applied to compare the alignment of the *GJB2* protein between different species.

### Literature review

We identified the relevant studies by searching the PubMed and Embase databases using relevant keywords and related spelling of “dominant” and “GJB2” on Feb 24, 2016. **Inclusion criteria:** i. primary source; and ii. report of *GJB2* variation in human subjects with NSHL. **Exclusion criteria:** i. patient population described specifically as having hearing loss acquired as a result of known prenatal, perinatal or postnatal ototoxic insult due to viral infection, jaundice, meningitis, or other conditions; and ii. sample population reported in a previous publication.

## Additional Information

**How to cite this article**: Wang, H. *et al*. A novel dominant *GJB2* (DFNA3) mutation in a Chinese family. *Sci. Rep.* **7**, 34425; doi: 10.1038/srep34425 (2017).

**Publisher's note:** Springer Nature remains neutral with regard to jurisdictional claims in published maps and institutional affiliations.

## Supplementary Material

Supplementary Information

## Figures and Tables

**Table 1:** Summary of clinical data for the hearing-impaired members of Family 304.

Subject	Gender^a^	Age of test (year)	Age of onset (year)	PTA (dB HL)^b^	Hearing impairment^c^	Audiogram	Tinnitus	Vertigo	Mutation^d^ detected
III:2	F	57	Not clear	L:71.25	Severe	Downslope	+	−	c. 524C > A
				R:67.5	Moderately severe	Downslope			
III:3	M	60	N/A	L:32.50	Mild	Downslope	−	−	−
				R:30.00	Mild	Downslope			
III:8	F	50	23	L:76.25	Severe	Downslope	+	−	c. 524C > A
				R:77.50	Severe	Downslope			
III:10	F	49	21	L:75.00	Severe	Downslope	+	−	c. 524C > A
				R:98.75	Profound	Downslope			
III:5	M	55	N/A	L:32.50	Mild	U-shape	−	−	−
				R:42.50	Moderate	U-shape			
III:12	F	47	0	L:>100	Profound	Flat	+	−	c. 524C > A
				R:66.25	Moderately severe	Downslope			
III:14	F	44	42	L:21.25	Normal	U-shape	−	−	−
				R:21.25	Normal	U-shape			
III:16	F	43	0	L:77.50	Severe	Downslope	−	−	c. 524C > A
				R:81.25	Severe	Downslope			
III:18	F	39	0	L:68.33	Moderately severe	Downslope	+	−	c. 524C > A
				R:>100	Profound	Flat			
IV:2	M	35	Not clear	L:78.75	Severe	Downslope	+	−	c. 524C > A
				R:90.00	Severe	Downslope			
IV:3	F	30	Not clear	L:53.75	Moderate	Downslope	+	−	c. 524C > A
				R:50.00	Moderate	Downslope			
IV:6	M	30	N/A	L:38.75	Mild	U-shape	−	−	-
				R:40.00	Mild	U-shape			
IV:12	M	24	0	L:72.50	Severe	Downslope	+	−	c. 524C > A
				R:73.75	Severe	Downslope			
IV:13	F	24	Not clear	L:43.75	Moderate	Downslope	+	−	c. 524C > A
				R:53.75	Moderately severe	Downslope			
IV:20	F	2	0	L:110dBnHL	Profound	/	−	−	c. 524C > A
				R:90dBnHL	Profound	/			
IV:21	F	14	0	L:70.00	Moderately severe	Downslope	+	−	c. 524C > A/c.235delC
				R:71.25	Severe	Downslope			

N/A, not available; +, positive finding; −, negative finding.

^a^M, male; F, female.

^b^PTA, pure-tone air-conduction averages (0.5, 1, 2 and 4 kHz). L, left ear, R, right ear. For the IV:20, who was 2 years old, her thresholds were the results of 40Hz AERP (auditory event-related potential) by tone burst.

^c^Diagnosed at the time of test.

^d^RefSeq: NM_004004, GRCh37/hg19 chr13.

**Table 2:** Overview of DFNA3 mutations identified to date.

No.	Mutation detected		Protein domain^b^	Origin	Phenotype	Families	Patients	Hearing impairment		Reference
	Nucleotide	Amino acid						Age of onset	PTA	
1	c.61G > A	p.G21R		Cuban	NSHL	1	/	Prelingual	Profound	Rabionet R, 2006
2	c.101T > C	p.M34T^a^		Caucasian	NSHL/SHL	1	6	/	Mild or profound	Kelsell DP, 1997
3	c.132G > C	p.W44C	EC1	USA	NSHL	1	3	Prelingual	Severe to profound	Tekin M, 2001
				France,	NSHL	2	20	Prelingual	/^b^	Denoyelle, F, 1998
4	c.131G > C	p.W44S	EC1	/	NSHL	/	/	/	/	Marziano NK, 2003
5	c.138T > G	p.D46E	EC1	Korea,	NSHL	1	4	Postlingual	Moderate	Choi SY, 2009
6	c.136G > A	p.D46N	EC1	Iran,	NSHL	2	10	Prelingual/postlingual	Severe to profound	Bazazzadegan N, 2011
7	c.164C > A	p.T55N	EC1	Italy,	NSHL	1	6	Postlingual	Profound	Melchionda S, 2005
8	c.172C > G	p.P58A	EC1	India,	NSHL	1	1	/	/	Primignani P, 2009
				Italy	NSHL	1	1	Prelingual	Profound	Primignani P, 2007
9	c.224G > A	p.R75Q	EC1	Korea,	NSHL	1	2	/	Moderate to severe	Kim J, 2015
				India,	NSHL/SHL	1	3	/	Profound	Pavithra A, 2015
				Ashkenazi	NSHL	1	5	Prelingual/postlingual	Moderate to profound	Sokolov M, 2014
				China	SHL	3	5	Prelingual/postlingual	Mild to profound	Pang XH, 2014
				Taiwan,	NSHL	1	4	/	Profound	Wu CC, 2013
				Brazil,	SHL	1	3	/	/	Manzoli GN, 2013
				Brazil,	NSHL	1	7	/	/	Manzoli GN, 2013
				Italy,	NSHL	1	2	Postlingual	Moderate	Iossa S, 2010
				German,	NSHL	2	3	Prelingual	/	Birkenhäger R, 2010
				France,	SHL	1	3	Postlingual	Mild to moderate	Feldmann D, 2005
				France,	NSHL	1	1	/	Moderate to profound	Feldmann D, 2005
				Turkey,	SHL	1	4	Prelingual/postlingual	Mild to severe	Uyguner O, 2002
10	c.223C > T	p.R75W	EC1	China,	SHL	2	4	Prelingual	Profound	Pang XH, 2014
				Netherlands,	NSHL	1	1	/	Severe to profound	Weegerink NJ, 2011
				Korea,	SHL	1	3	Prelingual	Severe to profound	Lee GY, 2010
				German	SHL	1	1	Prelingual	/	Birkenhäger R, 2010
				India,	NSHL	2	2	/	/	Mani RS, 2009
				China	SHL	1	1	Prelingual	Profound	Yuan YY, 2009
				Spain,	NSHL	1	1	/	Profound	Dalamón V, 2005
				Austria,	NSHL	1	1	Prelingual	Profound	Janecke AR, 2001
				Egypt,	SHL	1	2	Prelingual	Profound	Richard G, 1998
11	c.428G > A	p.R143Q	TM3	Tunisian	NSHL	1	2	Prelingual	Profound	Riahi Z, 2013
				China	NSHL	2	3	Prelingual	Severe to profound	Huang S, 2013
				Austria,	NSHL	1	5	Prelingual	Profound	Löffler J, 2001
12	c.487A > G	p.M163V	EC2	Iran,	NSHL	2	4	Prelingual/postlingual	/	Falah M, 2012
13	c.487A > C	p.M163L	EC2	Portuguese	NSHL	1	2	/	Mild/moderate	Matos TD, 2008
14	c.511G > A?	p.A171T	EC2	China	NSHL	1	/	Prelingual	Mild to severe	Xiao ZA, 2004
				USA	NSHL	1	1	/	Profound (unilateral)	Lin D, 2001
**15**	**c.524C > A**	**p.P175H**	**EC2**	**China**	**NSHL**	**1**	**8**	**Prelingual/postlingual**	**Moderate to profound**	**This study**
16	c.535G < A	p.D179N	EC2	India,	NSHL	4	4	/	/	Primignani P, 2009
				Italy,	NSHL	1	4	Postlingual	Mild to moderate	Primignani P, 2003
17	c.551G > A	p.R184Q	EC2	China,	NSHL	1	1	Prelingual	Profound	Pang XH, 2014
				China,	SHL	1	1	Prelingual	Severe	Pang XH, 2014
				Slovakia,	NSHL	1	1	/	/	Mina´rik G, 2012
				Mexico,	NSHL	1	1	/	Profound	Arenas-Sordo L, 2012
				Netherlands,	NSHL/SHL	1	2	/	Severe to profound	Weegerink NJ, 2011
				China	NSHL	2	2	Prelingual	Severe to profound	Huang S, 2011
				Iran,	NSHL	1	2	Prelingual	Severe	Mahdieh N, 2010
				India,	NSHL	1	1	/	/	Primignani P, 2009
				Taiwan,	NSHL	1	1	/	/	Wang YC, 2002
				Ghana,	NSHL	1	1	Prelingual	Profound	Hamelmann C, 2001
18	c.605G > T	p.C202F	TM4	French	NSHL	1	15	Postlingual	Mild to moderate	Morlé L, 2000
19	c.604T > C	p.C202R	TM4	Iran	NSHL	1	1	/	/	Onsori H, 2014
