# Supplementary material for: A novel dominant GJB2 (DFNA3) mutation in a Chinese family
Source: Sci Rep. 2017 Jan 19;7:34425. doi: 10.1038/srep34425 (PMC5244381; doi:10.1038/srep34425)
Supplement: Supplementary Information [file srep34425-s1.pdf]

A novel dominant GJB2 (DFNA3) mutation in a Chinese family

Hongyang Wang, Kaiwen Wu, Lan Yu, Linyi Xie, Wenping Xiong, Dayong Wang, Jing Guan, Qiuju Wang*

Institute of Otolaryngology, Chinese PLA General Hospital, Medical School of Chinese PLA, Beijing, 100853, China.

Corresponding author

Email: *wqcr@263.net.

Supplementary Figure S1. The ABR results of the propositus.

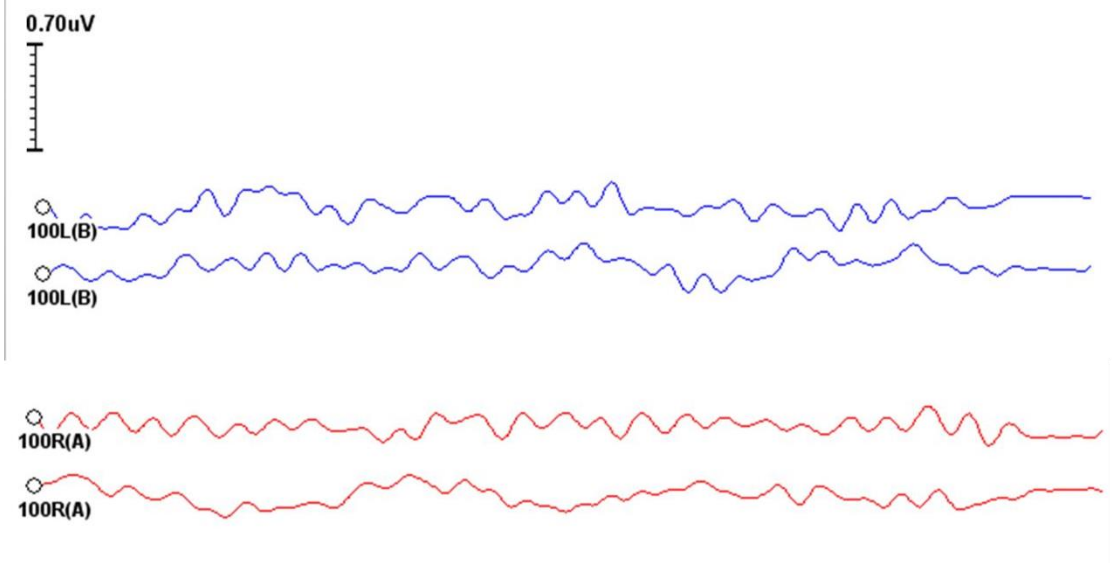

Supplementary Figure S2. Photos of the propositus showing absence of skin symptoms.

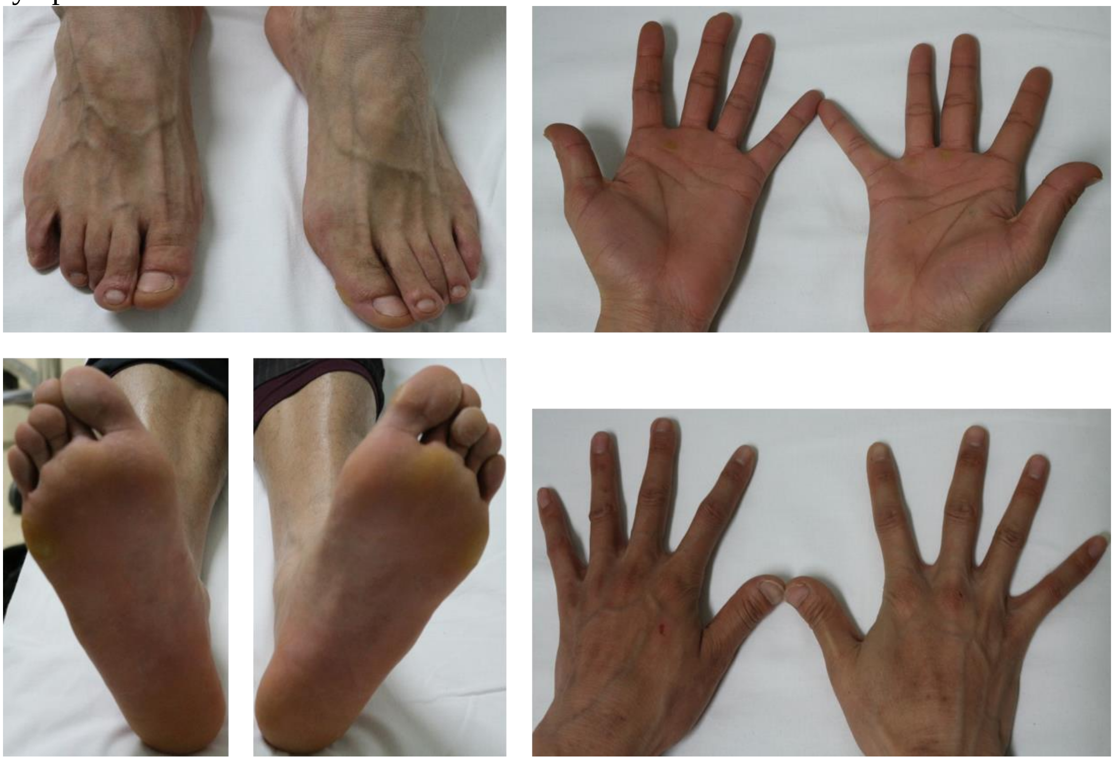

Supplementary Figure S3. DNA sequence chromatogram showing the heterozygous missense mutations c.524C>A in affected individuals and the c.235delC in IV:21.

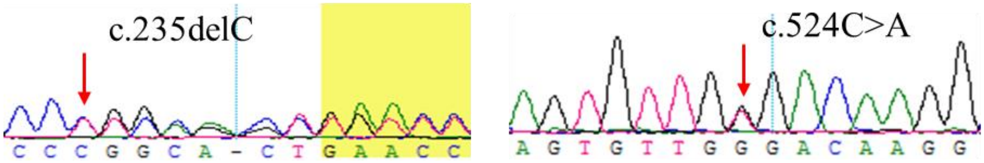

Supplementary Figure S4. Conservation analysis shows that the Pro residue at 175 in *GJB2* is conserved across.

Human	Q R L V K C N A W **P** C P N T V D C	175
Ptroglodytes	Q R L V K C N A W **P** C P N T V D C	175
Mmulatta	Q R L V K C N A W **P** C P N T V D C	175
Fcatus	Q R L V K C N A W **P** C P N T V D C	175
Mmusculus	Q R L V K C N A W **P** C P N T V D C	175
Xtropicalis	Q R L V Q C S N W **P** C P N V V	174
Ggallus	P R L M K C S A W **P** C P N T V D C	175
Drerio	A R L V K C E Q W **P** C P N K V	177

Supplementary FigureS5. The primer sequences and PCR cycle of *GJB2* gene.

Primer information of GJB2 EXON2: Product length 960bp

GJB2-Exon2- F：5′- TTGGTGTTTGCTCAGGAAGA-3′

GJB2-Exon2- R：5′- GGCCTACAGGGGTTTCAAAT-3′

GJB2 PCR Cycle: (S1000 PCR Instrument-BIO-RAD)

94℃，5min

1 cycle

94℃，30sec

63-58℃，40sec

72℃，40sec

10 cycles

94℃，30sec

58℃，40sec

72℃，40sec

27 cycles

72℃，5 min

4℃， F

1 cycle
